# TROP2: as a promising target in lung cancer

**DOI:** 10.3389/fonc.2025.1569897

**Published:** 2025-08-27

**Authors:** Xing-xing Li, Jia-li Chen

**Affiliations:** ^1^ Department of Medical Oncology, The First People’s Hospital of Linping, Hangzhou, Zhejiang, China; ^2^ Department of Pathology, The First People’s Hospital of Linping, Hangzhou, Zhejiang, China

**Keywords:** TACSTD2, TROP2, antibody-drug conjugate, lung cancer, lung adenocarcinoma

## Abstract

Lung cancer (LC) is a significant global health concern, underscoring the need for ongoing research into novel therapeutic modalities. Trophoblast cell surface antigen-2 (TROP2) is overexpressed in tumor tissues and minimally expressed in normal tissues, making it a promising target for cancer treatment. A TROP2-targeted antibody-drug conjugate (ADC) has been approved by the Food and Drug Administration (FDA). This review aims to provide a comprehensive overview of the characteristics of TROP2 and its role in cancer development. It is imperative to acknowledge the significant advancements made in the realm of LC therapy through the development of ADCs that specifically target the TROP2 antigen. The potential of the TROP2-ADC in the treatment of LC is a subject of considerable promise, suggesting a promising future in the therapeutic management of this condition.

## 1 Introduction

Lung cancer (LC) is a neoplasm with high global morbidity and mortality, accounting for 12.4% of new cases and 18.7% of deaths worldwide (1). The substantial basic, economic, and social burden necessitates the urgent development of effective treatment programs. The advent of targeted therapy and immunotherapy has marked a paradigm shift in the therapeutic landscape of LC (2). Nevertheless, the treatment of advanced LC patients with actionable genomic alterations and genetic mutations remains a formidable challenge. Intractable problems following include the lack of effective treatments after first-line treatment resistance, including the limited efficacy of back-line chemotherapy, chemotherapy combined with immunotherapy, and toxic side effects (3). To address these challenges, there is an urgent need to promote the development of novel drugs and treatment options.

Trophoblast cell surface antigen-2 (TROP2) is expressed at high levels in a variety of solid cancer cells and has been shown to affect signaling pathways involved in cancer proliferation, migration, invasion, and metastasis (4). TROP2 exhibits frequent overexpression across major histological subtypes of lung cancer, with particularly high prevalence observed in squamous cell carcinoma (approximately 60%), and adenocarcinoma (42%-64%) (5, 6). However, the responsiveness to TROP2 targeted therapeutics is depend on lung cancer subtype, rather than the expression of TROP2 (5). Squamous lung cancer appears to respond better to targeted TROP2 therapy than adenocarcinoma lung cancer. Therefore, TROP2 can be regarded as a viable target for the treatment of lung cancer, particularly in the context of NSCLC (7). This review will focus on the relationship between TROP2 and cancers, emphasizing the development of TROP2-ADC.

## 2 Structure and function of TROP2

**Figure 1:**
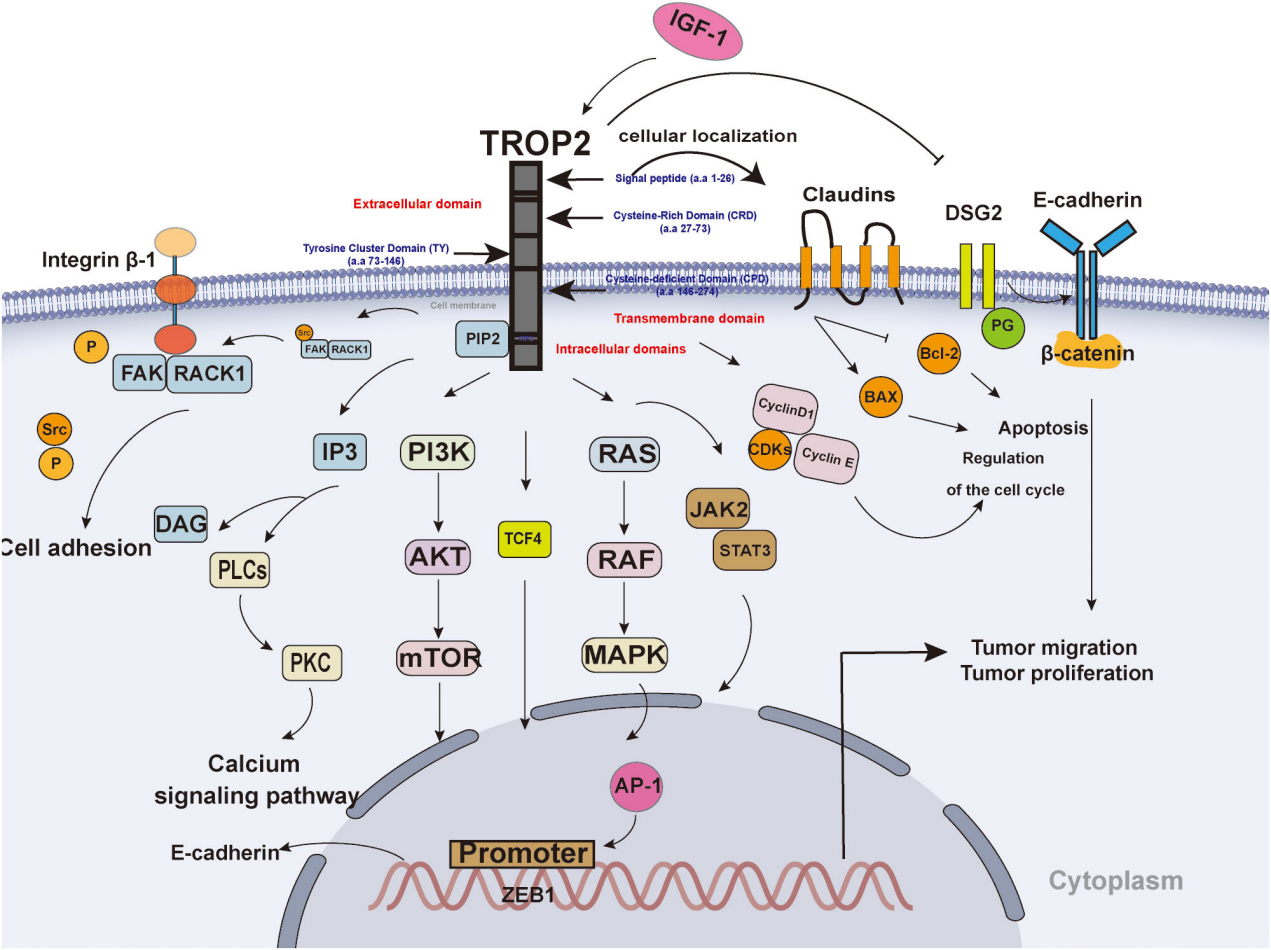
The Structure and signaling pathway of TROP2. TROP2 consists of an extracellular domain, a transmembrane domain, and an intracellular domain. TROP2 affects tumor proliferation and migration through multiple pathways: 1) TROP2 interacts with IGF-1)and affects downstream signaling, such as the PI3K-AKT and MAPK pathways; 2) TROP2 regulates the expression of cyclin D1, cyclin E, and CDK to promote the cell cycle; 3) TROP2 promotes the transition from PIP2 to IP3 and DAG;4) TROP2 promotes the recruitment of RACK1 and contributes to spatial coupling between FAK and β1-integrin; 5) TROP2 transcriptionally regulates ZEB1 expression, contributes to the expression of E-cadherin; 6) TROP2 decreases the expression level of DSG2 to promote tumor cell invasion and migration by EGFR-AKT and DSG2-PG-β-catenin pathways; 7) TROP2 downregulated Bcl-2 and upregulated Bax; 8) TROP2 promotes cell motility and claudin-7 localization to cellular borders; 0) TROP2 promote JAK2-STAT3 signaling pathway.

The TACSTD family comprises TACSTD1 and TACSTD2, two genes that are highly conserved and closely related, and respectively encode TROP2 and epithelial cell-adhesion molecule (EpCAM) (8). TROP2 functions as a single transmembrane protein comprising an extracellular domain (ECD), a single transmembrane domain, and a short cytoplasmic tail (7). The ECD contains a Cysteine-Rich Domain (CRD), a Tyrosine Cluster Domain (TY), and a Cysteine-deficient Domain (CPD), which collectively contribute to the formation of a stable dimer (**Figure 1**) (9, 10). TROP2 exhibits low expression levels in normal epithelial cells and high expression levels in many epithelial tumors (e.g., colon, pancreas, and breast) (**Figure 1**) (9). The ECD of TROP2 is anchored to the cell membrane by a unidirectional transmembrane helix (TM) attached to the intracellular structural domain (ICD) (**Figure 1**). The ECD of TROP2 affects signaling transduction by a conserved phosphatidylinositol-4,5-bisphosphate (PIP2) binding sequence (11). The intracellular structural domains contain sites that interact with a variety of signaling proteins, such as the phosphorylation site of protein kinase C (PKC) (12).

TROP2 has been shown to promote tumor cell proliferation by regulating the calcium signaling pathway, cell cycle protein expression and reducing fibronectin adhesion. TROP2 interact with insulin-like growth factor 1 (IGF-1) and its ligands to affect the upstream (**Figure 1**) (13). The S303 site of the intracellular domain of TROP2 undergoes phosphorylation, which in turn promotes a series of critical intracellular signals, including the transition from PIP2 to inositol 1,4,5-trisphosphate (IP3) and diacylglycerol (DAG) (14). This, in turn, has been shown to stimulate the mitogen-activated protein kinase (MAPK) signaling pathway and promote the activation of activator protein 1(AP-1) (**Figure 1**) (14). Furthermore, the activated MAPK signaling pathway has been associated with epithelial-mesenchymal transition (EMT), which in turn affects tumor invasion (15). The transcription factor AP-1 has been identified as a contributing factor to angiogenesis (16). TROP2 promote growth and proliferation through the activation of the janus kinase 2 (JAK2)- signal transducer and activator of transcription 3(STAT3) pathway (17). In addition, TROP2 upregulated B-cell lymphoma 2 (Bcl-2) and downregulated Bcl-2-associated X protein (Bax), promoting tumorigenesis and tumor progression (18). Further, TROP2 has been shown to regulate the expression of cell cycle proteins, including CyclinD1, CyclinE, and CDK proteins such as cyclin-dependent kinase 2 (CDK2) and CDK4, thereby promoting cell cycle progression (**Figure 1**) (19). Ser-322 by PKCα/phosphorylates TROP2 to promote cell motility and claudin-7 localization to cellular borders (20). Further, β-conjugate protein/transcription factor 4 (TCF4) has the capacity to bind to the C-terminal fragment of TROP2 to the promoter of zinc finger E-Box binding homeobox 1 (ZEB1), and upregulate of ZEB1 expression (21). As a result, The ZEB1 contributes to the expression of E-cadherin, and influence tumor aggressiveness and metastatic capacity (21). Besides, overexpression of TROP2 decreases the expression level of desmoglein 2 (DSG2), activates EGFR-AKT and DSG2-plakoglobin(PG)-β-catenin pathways to promote tumor cell invasion and migration (22). TROP2 upregulation promotes the membrane translocation of receptor for activated C kinase 1 (RACK1), thereby establishing spatial coupling between focal adhesion kinase (FAK) and β1-integrin (**Figure 1**) (23). This interaction triggers FAK activation through autophosphorylation at Tyr397, which releases Src from the complex and activates it (23). Ultimately, this leads to a reduction in tumor cell adhesion (**Figure 1**) (23).

## 3 TROP2-targeted therapeutics

**Figure 2:**
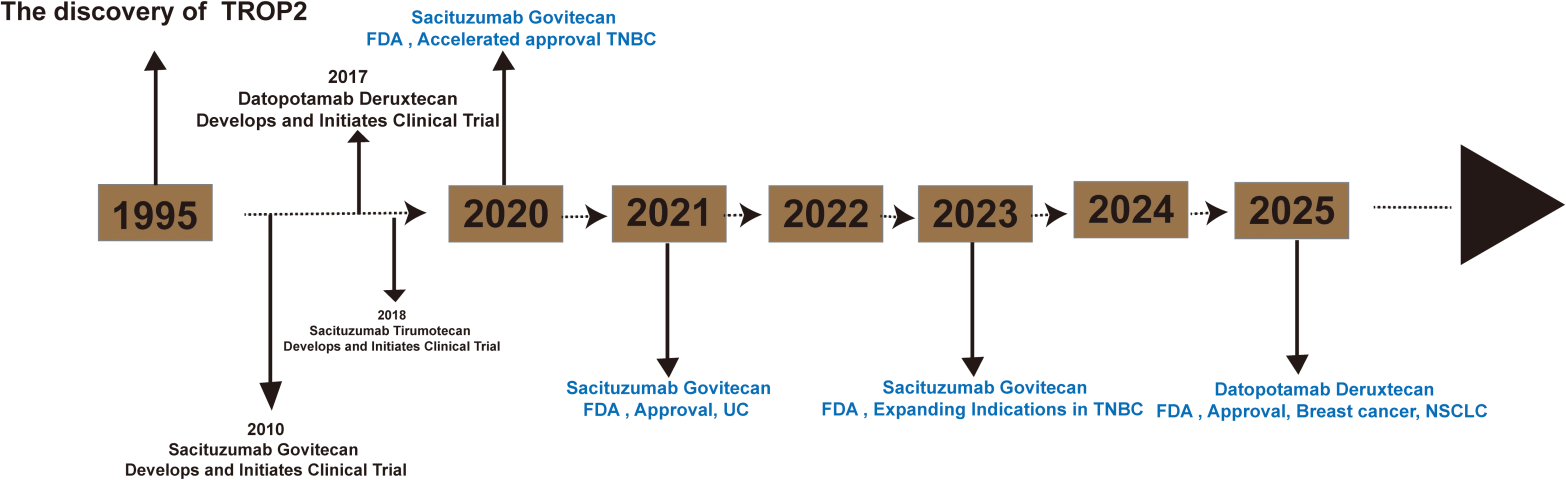
Timeline for the launch of TROP2-ADC (sacituzumab govitecan, datopotamab deruxtecan and sacituzumab tirumotecan).

TROP2-targeted therapeutics are predominantly formulated as ADC (24) (**Table 1**). ADCs represent a novel class of antitumor drugs that exhibit the high specificity of monoclonal antibodies and the high activity of small-molecule cytotoxic drugs (25). These drugs consist of antibodies, linkers, and payloads. Monoclonal antibodies are capable of recognizing targeted antigens on the surface of tumor cells. The ADC complex enters the cell interior via receptor-mediated endocytosis, releasing the cytotoxic drug (26). Furthermore, the diffusion of cytotoxic drugs released by ADC through the cell membrane can also lead to the destruction of neighboring cancer cells (27). The FDA has approved several ADCs targeting TROP2 for marketing (**Figure 2**).

**Table 1:** The clinical studies of Trop2-targeted therapeutics in lung cancer.

NCT number	Drug	Combination	Cateories	Phase	Current status
NCT05941507	LCB84	Anti-PD-1 monoclonal antibody	ADC	I/II	Recruiting
NCT06357533	Datopotamab Deruxtecan	Rilvegostomig,Pembrolizumab	ADC	III	Recruiting
NCT06454890	Anti-Trop2 CAR-NK cell		CAR-T	I/II	Not yet recruiting
NCT04152499	SKB264		ADC	I/II	Recruiting
NCT05687266	Datopotamab Deruxtecan		ADC	III	Active, not recruiting
NCT03401385	Datopotamab Deruxtecan	Steroid Containing Mouthwash,Non-Steroid Containing Mouthwash	ADC	I	Active, not recruiting
NCT06676917	Datopotamab Deruxtecan		ADC	II	Not yet recruiting
NCT05460273	Datopotamab Deruxtecan		ADC	I/II	Active, not recruiting
NCT05969041	MT-302 (A)		CAR	I	Recruiting
NCT04526691	Datopotamab Deruxtecan	KEYTRUDA,Carboplatin,Cisplatin	ADC	I	Active, not recruiting
NCT04940325	Datopotamab Deruxtecan		ADC	II	Active, not recruiting
NCT06564844	Datopotamab Deruxtecan	Rilvegostomig,Carboplatin	ADC	III	Recruiting
NCT04612751	Datopotamab Deruxtecan	Durvalumab,Carboplatin	ADC	Ib	Recruiting
NCT05865990	Patritumab deruxtecan		ADC	II	Active, not recruiting
NCT06350097	Datopotamab Deruxtecan	Osimertinib	ADC	III	Recruiting
NCT06417814	Datopotamab Deruxtecan	Osimertinib,Pemetrexed	ADC	III	Recruiting
NCT04656652	Datopotamab Deruxtecan	Docetaxel	ADC	III	Active, not recruiting
NCT06074588	Sacituzumab tirumotecan	Docetaxel,Pemetrexed	ADC	III	Recruiting
NCT06480136	SHR-A1921	Adebrelimab	ADC	II	Not yet recruiting
NCT05609968	Sacituzumab Govitecan	MK-3475	ADC	III	Recruiting
NCT06431633	Sacituzumab Govitecan	Zimberelimab,Cisplatin,Carboplatin	ADC	II	Not yet recruiting

### 3.1 Sacituzumab govitecan

Sacituzumab govitecan is an antibody-drug conjugate developed to treat solid tumors, including breast, lung, and urothelial cancers. It contains the irinotecan active metabolite SN-38 (govitecan) site-specifically conjugated to a humanized monoclonal antibody (hRS7) directed against the CPD domain of TROP2 (28). *In vitro*, sacituzumab govitecan exhibits a mechanism similar to that of free SN-38, and hRS7 shows potent broad anticancer activity in human cancer xenografts and in patients (28).

The combination of targeted drugs and chemotherapeutic drugs in sacituzumab govitecan (SG) represents a novel approach to cancer treatment. This combination has the potential to enhance the efficacy of targeted drugs in targeting tumor tissues while concurrently reducing the impact on normal tissues (29). Notably, SG is primarily target-oriented for patients diagnosed with triple-negative breast cancer (TNBC) (29, 30). The TROPiCS-02 study demonstrated that the combination of SG improved median overall survival (OS) (14.4 months *vs*. 11.2 months) and reduced overall risk of death by 11% (31). SG has been shown to demonstrate superior efficacy and safety in the second-line treatment of small cell lung cancer and non-small cell lung cancer, particularly in cases where patients have become resistant to conventional treatment regimens. In a subsequent TROPiCS-03 trial targeting extensive stage small cell lung cancer, the objective remission rate (ORR) was achieved at 41.9%, accompanied by a median progression-free survival (PFS) of 4.4 months and a median OS of 13.6 months (32). The clinic trial placed particular emphasis on the controlled security of SG in lung cancer (32). The efficacy of SG in the treatment of NSCLC is well-documented. A subsequent EVOKE-01 trial, which was designed to compare SG and chemotherapy, revealed that SG enhances OS, particularly among patients who are refractory to programmed cell death-ligand 1 (PD-L1) therapy, while concurrently ensuring enhanced manageable safety (33).

### 3.2 Datopotamab deruxtecan

Datopotamab Deruxtecan (Dato-DXd) is composed of the anti-TROP-2 IgG1 monoclonal antibody Datopotamab linked with the topoisomerase I inhibitor exatecan derivative (DXd) via a cleavable tetrapeptide linker, ensuring precise eradication of tumor cells (34). Dato-DXd suppresses the proliferation of TROP2-expressing tumors by recognizing the TROP2 ectodomain (ECD) in tumor cells. Furthermore, a study verifies that DXd exhibits more potent Topo inhibitory activity than SN-38 (35). The Dato-DXd combination has demonstrated notable antitumor activity across various tumor types, particularly in NSCLC and breast cancer (34, 36). The TROPION-Breast01 trial, which involved the utilization of Dato-DXd, demonstrated a substantial reduction in disease progression risk, with a median PFS of 6.9 months in comparison to the chemotherapy group (37).

Dato-DXd is currently engaged in testing and exploration in the domain of lung cancer. In the randomized, phase III, open-label, global study TROPION-Lung01, which enrolled 604 patients with lung cancer, the Dato-DXd treatment group demonstrated a significant increase in PFS (4.4 months *vs*. 3.7 months, HR=0.75, 95%Cl 0.62-0.91; P=0.004) when compared to the docetaxel group. In addition, the Dato-DXd group exhibited superior objective remission rate (ORR), median duration of remission (DOR), and disease control rate (DCR) (ORR: 26.4% *vs*. 12.8%, DOR: 7.1 months *vs*. 5.6 months, DCR: 77.3% *vs*. 64.9%) (38). It is anticipated that Dato-DXd will emerge as a potential treatment option for patients with NSCLC who have developed resistance to EGFR- tyrosine kinase inhibitors (TKIs). The TROPION-Lung05 study demonstrated that patients receiving the treatment of Dato-DXd achieved confirmed ORR of 35.8% and median DOR of 7.0 months (39). These findings offer a range of treatment options for patients with NSCLC who are eligible for Dato-DXd therapy. A multitude of clinical trials are currently being conducted on the subject of Dato-DXd, aimed to explore the possibility of combination (**Table 1**). Consequently, the FDA successively approved Dato-DXd in 2025 for the treatment of hormone receptor (HR)-positive/human epidermal growth factor receptor2 (HER2)-negative breast cancer and locally advanced or metastatic epidermal growth factor receptor (EGFR)-mutated NSCLC.

### 3.3 Sacituzumab tirumotecan (SKB264)

The shedding of the SN-38 payload from SG leads to off-target toxicity, including neutropenia, diarrhea, vomiting, and nausea (40). Sacituzumab Tirumotecan is a humanized IgG1 mAb hRS7 conjugated with a property cytotoxic developed using a novel DNA topoisomerase I inhibitor (KL610023) and optimized ligation methods to address off-target toxicity (41, 42). In TROP2-expressing xenograft models, sacituzumab tirumotecan demonstrates strong efficacy, a favorable safety profile, and an excellent therapeutic window (40).

Sacituzumab tirumotecan is a class of TROP2-ADC that is currently undergoing clinical trials for various cancers (**Figure 1**, **Table 1**). In a phase I/II clinical trial recruiting EGFR wild and EGFR mutation patients, the ORR was 40%, with a median PFS of 6.2 months and the efficacy benefit was even more prominent in EGFR mutant patients compared to EGFR wild-type patients (43). The efficacy of the drug has been demonstrated in patients with EGFR mutation lung cancer. In a phase II clinical trial designed to assess the efficacy of Sacituzumab tirumotecan in patients resistant to EGFR-TKI who received platinum-containing chemotherapy, the study cohort treated with sacituzumab tirumotecan achieved an ORR of 34%, a median PFS of 9.3 months (43). In a clinical trial designated as OptiTROP-Lung01, patients were administered Sacituzumab tirumotecan in combination with KL-A167, a PD-L1 inhibitor. The study observed an ORR of 59.3%, accompanied by manageable safety profiles (44).

### 3.4 Next-generation of TROP2-targeted therapeutics

Current developments focus on novel TROP2-targeted therapeutics, including dual antibody ADCs, bispecific T cell engager (BiTE) antibodies, and a TROP2-targeted nano-in-gel vaccine (NIGel-Vax). However, dual antibody ADCs present significant challenges. A minimal dose must be verified for each payload to obtain a response, and stable molecular structures are essential (45). BiTEs are a new type of immunotherapy that recognizes both tumor surface antigens and CD3. F7AK3, a bispecific antibody targeting TROP2 and CD3, has demonstrated remarkable antitumor efficacy *in vitro* and *in vivo* (46). Additionally, a novel bispecific antibody was designed by reducing the binding affinity of CD3 in two steps to reduce its ability to stimulate cells (47). Nectin cell adhesion protein 4 (Nectin-4), a member of the Nectin family, is specifically expressed in tumor tissues (48). Clinical trials for a Nectin-4/TROP2 dual antibody ADC are currently in development. A phase 1 clinical study is evaluating the safety, tolerability, pharmacokinetics (PK), immunogenicity, and preliminary antitumor efficacy of AK146D1 in patients with advanced solid tumors (NCT06929663). NIGel-Vax, a novel cancer immunotherapy, exerts anti-tumor effects by stimulating T cells and increasing memory T cells, among other immune amplifications (49). In TNBC models, NIGel-Vax targeting TROP2 achieved a 96% tumor suppression rate and a 50% cure rate (49).

## 4 The resistance mechanism of TROP2-ADC

Although TROP2-targeting ADCs have demonstrated remarkable clinical efficacy, drug resistance continues to pose a significant therapeutic challenge. However, research on the mechanisms underlyingTROP2-ADC resistance remains limited. Reduced target (TROP2) expression represents a common mechanism of resistance to TROP2-targeting ADCs. Exposure to the treatment of ADCs, tumor cells experiencing a marked decrease in antigen levels shortly (50). Therefore, the also points to the downregulation of TROP2 in treatment of TROP2-ADC. For instance, a triple-negative breast cancer patient lacking TROP2 expression exhibited *de novo* resistance to SG (51). Additionally, acquired resistance to SG (IMMU-132) in another patient was associated with a mutated TROP2 protein, leading to reduced ADC binding due to altered subcellular localization (52).

Efflux of the ADC payload constitutes another resistance mechanism. The payload SN-38, released from SG, is a representative topoisomerase I inhibitor. However, its unfavorable physicochemical properties—such as poor solubility and stability—hinder effective delivery to tumor sites (53). Furthermore, the upregulation of multi-drug resistance (MDR) pathways and tumor heterogeneity contribute to both inherent and acquired resistance to the SN-38 payload (54). To address this resistance, researchers have developed a novel class of ADCs utilizing frontal T moiety-exatecan conjugates, demonstrating efficacy without significant toxic side effects (54).

Furthermore, the lysosomal degradation of ADCs depends on an acidic lysosomal environment and the activity of lysosomal enzymes (55). Lysosomal dysfunction can therefore impair ADC efficacy. For example, in the enmetuximab (T‐DM1)-resistant breast cancer cell line BT474, researchers observed lysosomal alkalization and impaired activity of lysosomal proteolytic enzymes (56). Additionally, beyond lysosomal dysfunction, impaired expression of Endophilin A2 (Endo II) in HER2-positive (HER2+) breast cancer models reduced HER2 internalization and diminished the response to T-DM1 (57). As a scaffolding protein, Endo II plays a role in clathrin-independent endocytosis. In conclusion, internalization and lysosomal dysfunction also leads to drug resistance problems.

## 5 Conclusion and prospective

TROP2, a glycoprotein, has been shown to regulate a variety of pathological activities, including tumor growth and migration. Its distinct expression in various tumors renders it an optimal target for multiple therapeutic interventions. The elevated expression levels of TROP2 in tumors suggest that targeted therapeutic agents are more likely to bind to tumor cells, thereby enhancing the therapeutic effect and reducing adverse effects. The field of oncology has witnessed significant advancements in the development of TROP2 population particularly in the context of lung cancer and breast cancer. Emerging evidence suggests that TROP2-targeted therapeutic strategies may demonstrate clinical benefits in cancer patients regardless of detectable TROP2 expression status. However, the therapeutic targeting of TROP2 is confronted with significant challenges, including off-target toxicity and acquired resistance. To address these limitations, promising strategies involve optimizing ADC design, developing next-generation ADCs with enhanced tumor selectivity, and exploring synergistic combination therapies with immune checkpoint inhibitors or targeted agents. Concurrently, an increasing number of clinical trials are being developed to expand the utilization of these targeted drugs to various tumor populations. As a class of drugs specifically targeted to tumors, TROP2-targeted therapeutics are widely regarded as poised to transform the future of solid tumor therapy, such as gastric cancer, pancreatic cancer, breast cancer, prostate cancer (58–61).
